# Pilot study evaluating the efficacy of a topical formulation containing emodepside and praziquantel in the treatment of natural feline troglostrongylosis

**DOI:** 10.1186/s13071-019-3361-7

**Published:** 2019-03-12

**Authors:** Donato Traversa, Fabrizia Veronesi, Patrizia Danesi, Simone Morelli, Paolo E. Crisi, Giulia Morganti, Raffaella Iorio, Fabrizio Pampurini, Roland Schaper, Azzurra Santoro, Barbara Paoletti, Angela Di Cesare

**Affiliations:** 1 Faculty of Veterinary Medicine, University of Teramo, Teramo, Italy; 2 Department of Veterinary Medicine, University of Perugia, Perugia, Italy; 3 Istituto Zooprofilattico Sperimentale delle Venezie, Legnaro, Padua Italy; 4 Bayer Animal Health, Milano, Italy; 5 Bayer Animal Health GmbH, Leverkusen, Germany

**Keywords:** *Troglostrongylus brevior*, Cat, Emodepside, Treatment

## Abstract

**Background:**

*Troglostrongylus brevior*, a lungworm usually affecting wild felids, has been recently recorded in a number of cases in domestic cats, mainly in Mediterranean areas. Although feline troglostrongylosis is a severe and life-threatening disease, especially in young cats, treatment options are very limited. The present study evaluated the efficacy and safety of a spot-on formulation containing emodepside 2.1% and praziquantel 8.6% (Profender^®^, Bayer), which is licensed for treatment of the more common cat lungworm *Aelurostrongylus abstrusus*, for the treatment of natural troglostrongylosis.

**Methods:**

Sixteen cats enrolled in the study were 1:1 allocated to two groups, i.e. Group T, treated with Profender^®^ spot-on on days 0 and 14 (± 2) at the recommended clinical dose, and Group C which remained untreated. After study completion, the control cats received two rescue treatments with Profender^®^ on days 28 (± 2) and 42 (± 2). The primary efficacy criterion was the absence of *T. brevior* L1 following treatment. Other efficacy parameters were the quantitative comparison of L1 presence before (baseline) and after treatment in both groups, and the comparison of clinical signs pre- and post-treatment.

**Results:**

In terms of stopping larval shedding, Profender^®^ showed an efficacy of 97% and 97.5% (arithmetic and geometric means, respectively) for group T, 97.1% and 98.5% for group C after one administration, and 100% for both groups after two doses. Overall, 12 cats showed clinical signs related to *T. brevior*. Specifically, 9 were clinically affected before treatment while clinical signs appeared after the first treatment in 3 cats. At the end of the study, all symptomatic cats fully recovered with the exception of 3 cats that showed clinical signs similar to those observed at the pre-treatment examination at the end of the study.

**Conclusions:**

This study shows that Profender^®^ is effective against *T. brevior*.

## Background

Feline troglostrongylosis is an emerging gastropod-borne disease caused by the metastrongyloid lungworm *Troglostrongylus brevior* [1]. This parasite usually affects wild felids but recently several cases of infection in domestic cats have been described [2]. Adult stages live in bronchi and bronchioles and, after mating, females produce eggs that hatch releasing first-stage larvae (L1), which migrate to the pharynx, where they are swallowed and then shed in feces [1, 3–5]. The life-cycle of *T. brevior* is similar to that of the globally distributed and well-known cat lungworm *Aelurostrongylus abstrusus* and cats become infected by ingesting third-stage larvae (L3) in intermediate hosts, i.e. slugs and snails, or paratenic hosts, i.e. rodents, amphibians, birds and reptiles [3, 4]. Additionally, a vertical route of transmission has been described [6–9]. Although it is not definitively proven how *T. brevior* is vertically transmitted, it is likely that the infection takes place in the first days after birth, likely *via* the colostrum or milk [8].

Thus far, feline troglostrongylosis has been mainly recorded in geographical areas where the natural host, i.e. the European wildcat, is present, e.g. Italy, Spain, Greece, Bulgaria [2, 9, 10]. The clinical picture in cats infected with *T. brevior* is characterized by respiratory distress (i.e. cough, dyspnea, polypnea, nasal discharge, irreversible pulmonary hypertension), and non-respiratory signs (i.e. anorexia, dehydration, poor general condition, depression) [4, 9, 11, 12]. The severity of clinical signs is greater in younger cats and, especially in animals only a few weeks- or months-old, the infection is life-threatening [2, 4, 7, 8, 13].

Despite the major pathogenic role of *T. brevior*, very few therapeutic options are available. Thus far, the only licensed product is a spot-on formulation containing eprinomectin 0.4% in combination with fipronil 8.3%, (S)-methoprene 10% and praziquantel 8.3% (Broadline^®^, Merial-Boehringer Ingelheim). In field conditions this product has shown an efficacy of up to 100% [10, 14], but it should be taken into account that, when specified by the SPC (summary of product characteristics) it is exclusively indicated when cestodes, nematodes and ectoparasites are to be targeted at the same time.

The spot-on formulation containing emodepside 2.1% and praziquantel 8.6% (Profender^®^, Bayer) is efficacious for treating natural [15] and experimental [16] infections caused by the cat lungworm *A. abstrusus*, and it has recently been labelled for this purpose in the European Union. Furthermore, its efficacy against *T. brevior* showed to be promising in individual clinical cases, even in mixed infections caused by other respiratory nematodes [8, 12, 13]. The present pilot study has evaluated for the first time the efficacy of Profender^®^ in the treatment of feline troglostrongylosis in a case series of naturally infected animals.

## Methods

The study was a blinded, randomized, negative-controlled field trial carried out at three sites located in endemic areas of the Umbria (Site A) and Abruzzo (Sites B and C) regions of central Italy.

### Pre-inclusion screening

**Fig. 1:**
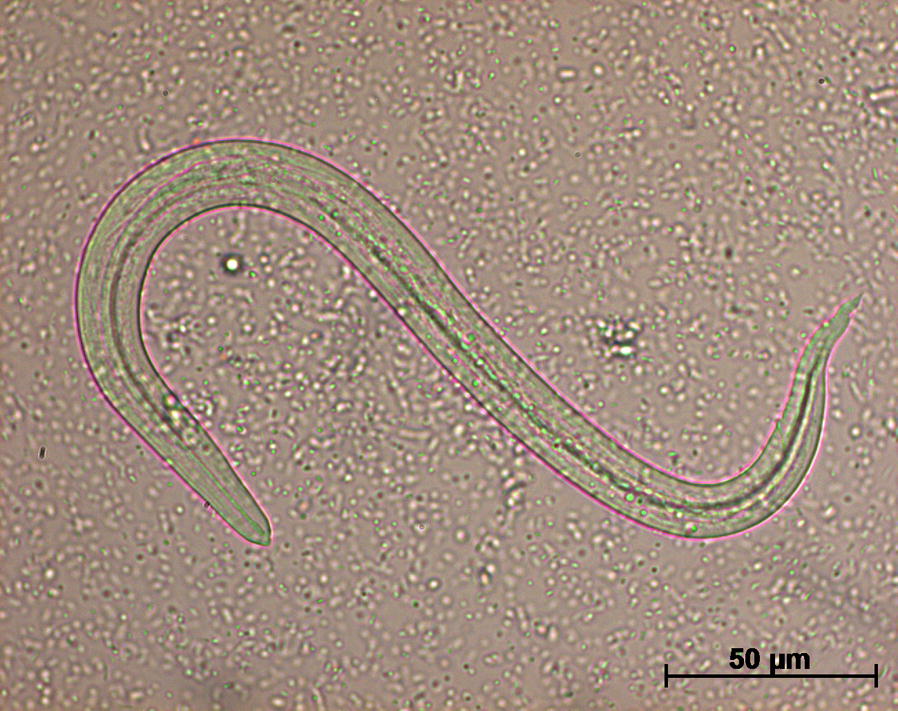
First-stage larva of *Troglostrongylus brevior*

Privately owned cats were enrolled upon informed consent signed by the owner. Individual fecal samples from 165 cats, i.e. 85, 54 and 26 from sites A, B and C, respectively, were collected and tested using the Baermann migration method for the presence of *T. brevior* L1 on days -30/-7. Larvae were identified as *T. brevior* (Fig. 1) according to morphometric and morphological features [4, 5] and their identity was genetically confirmed using species-specific PCR [7]. A total of 16 (9.7%) positive cats, 9 (10.6%), 5 (9.3%) and 2 (7.7%) from sites A, B and C, respectively, were included in the study according to inclusion/exclusion criteria.

### Inclusion and exclusion criteria

Cats were enrolled in the study according to the inclusion/exclusion criteria as follows. Inclusion criteria: (i) cats in good general health for which the owner signed the owner consent form; (ii) copromicroscopic detection of L1 of *T. brevior* in at least one Baermann examination performed between days -30 and -7, whose identity has been confirmed by PCR. Exclusion criteria: (i) cats treated with a macrocyclic lactone or other anthelmintics with a systemic biodistribution within 2 months before the study; (ii) cats affected by concomitant parasitic respiratory infections; (iii) cats less than 8 weeks-old; (iv) cats weighing less than 0.5 kg; (v) pregnant or lactating cats; (vi) animals with severe clinical signs of troglostrongylosis or suffering from other health conditions.

All 16 cats fulfilled the inclusion criteria and on days -7/0 were clinically and copromicroscopically examined with a quantitative Baermann test to assess values of *T. brevior* larvae per gram of feces (LPG).

### Treatment and post-treatment evaluation

On day 0 each cat underwent a clinical examination, was weighed and randomly assigned to the treatment (T) or to the control (C) Group. Cats of Group T (*n* = 8 cats) were treated with Profender^®^ spot-on on days 0 and 14 (± 2) at the recommended clinical dose, while cats of Group C remained untreated and, after study completion, received two rescue treatments on days 28 (± 2) and 42 (± 2).

Larval counts were again performed post-treatment on days 14 (± 2) and 28 (± 2) for Group T and on days 42 (± 2) and 56 (± 2) for Group C. On day 0 and the same day of each copromicroscopic test all cats underwent a physical examination to evaluate the presence of clinical signs associated with troglostrongylosis. The cats were also observed daily by their owners for the entire period of the study.

### Clinical examination

Clinical procedures were performed by the veterinarian in charge for each of the study sites. The presence of respiratory distress and other signs (listed in Table 2) was documented on a clinical examination form using an evaluation grid based on scores for each change in order to provide parameters that were as objective as possible. A total clinical score was calculated for each cat based on the sum of the different scores on days 0, 14 (± 2) and 28 (± 2) (Group T) and 28, 42 (± 2) and 56 (± 2) (Group C).

### Efficacy evaluation

The primary efficacy criterion was the presence/absence of *T. brevior* after treatment. The presence of the nematode was defined on day 28 (± 2) (post-treatment) according to the detection of L1 in Baermann tests of Group T.

The following efficacy criteria were also evaluated:

(i) Statistical comparison of the LPG values between pre-treatment (baseline) and post-treatment copromicroscopic evaluations within Group T, and between the pre-treatment and post-treatment copromicroscopic evaluations within Groups T and C, according to the formula:
  $$\% \;{\text{reduction}} = {{\left( {{\text{Mean}}\;{\text{LPG}}\;{\text{at}}\;{\text{baseline}}-{\text{Mean}}\;{\text{LPG}}\;{\text{at}}\;{\text{post-baseline}}} \right)} \mathord{\left/ {\vphantom {{\left( {{\text{Mean}}\;{\text{LPG}}\;{\text{at}}\;{\text{baseline}}-{\text{Mean}}\;{\text{LPG}}\;{\text{at}}\;{\text{post-baseline}}} \right)} {{\text{Mean}}\;{\text{LPG}}\;{\text{at}}\;{\text{baseline}} \times 100}}} \right. \kern-0pt} {{\text{Mean}}\;{\text{LPG}}\;{\text{at}}\;{\text{baseline}} \times 100}}$$
  where Mean LPG was calculated as arithmetic and geometric means.
(ii) Comparison of qualitative and quantitative presence of L1 before (baseline) and after the rescue treatment in Group C, according to the above formula.
(iii) Post-treatment clinical evaluation of clinically affected cats within Groups T and C, compared with pre-treatment clinical evaluations.

## Results

All cats included in the efficacy evaluation of Profender^®^ completed the study according to protocol and none of them showed any adverse event.

### Efficacy

#### Qualitative efficacy

Overall, 7 out of 8 cats from both Groups T and C (87.5%) were negative in the Baermann examination on days 14 (± 2) and 42 (± 2) after a single administration of Profender^®^, respectively.

The second administration of the drugs guaranteed cessation of larval excretion in the remaining two cats (100%), i.e. 1 from group T on day 28 (± 2) and 1 from group C on day 56 (± 2).

#### Quantitative efficacy

At baseline, cats included in the study had an average of 118.1 LPG. The mean LPG of cats from Group T and C was 106.8 and 129.3, respectively (Table 1). The percentage of reduction for cats of Group T on days 14 (± 2) and 28 (± 2) was 97.0% and 97.5% (ANOVA, *F*_(8,8)_ = 9.470, *P* = 0.008) and 100% and 100% (arithmetic and geometric means) (ANOVA, *F*_(8,8)_ = 17.883, *P* = 0.001), respectively, while it was 97.1% and 98.5% on day 42 (± 2) (ANOVA, *F*_(8,8)_ = 17.905, *P* = 0.001) and 100% and 100% on day 56 (± 2) for cats of Group C (ANOVA, *F*_(8,8)_ = 19.298, *P* = 0.001) (Table 2).

**Table 1:** Homogeneity of the two study groups for LPG tested by analysis of variance (ANOVA) at baseline (*F*_(8,8)_ = 0.422, *P* = 0.527)

Group	*n*	Mean LPG	SD	SE	95% CI	Min	Max
T	8	106.8	51.612	18.248	63.73–150.02	45	195
C	8	129.3	83.300	29.451	59.73–199.02	15	225
Total	16	118.1	67.943	16.986	81.92–154.33	15	225

*Abbreviations*: CI, confidence interval; Min, minimum; Max, maximum; SD, standard deviation; SE, standard error

**Table 2:** Arithmetic and geometric means calculated for LPG of the two groups at each fecal collection. Calculation of geometric mean was done on data transformed in (LPG + 1)

Group	Day	Mean	SD	SE	Min	Max	Geometric mean
T	G07	106.8	51.61	18.25	45	195	97.0
	G14	3.7	10.61	3.75	0	30	1.5
	G28	0	0	0	0	0	0
C	G07	129.3	83.30	29.45	15	225	100.3
	G14	125.0	110.94	39.22	0	300	61.7
	G28	102.5	68.56	24.24	15	225	79.4
	G42	3.7	10.61	3.75	0	30	1.5
	G56	0	0	0	0	0	0

*Abbreviations*: Min, minimum; Max, maximum; SD, standard deviation; SE, standard error

### Clinical outcome

On day 0, 6 out of 8 cats (nos. 3, 6, 7, 12, 13 and 16) of Group T showed clinical signs associated with troglostrongylosis (Table 3). Among them, 4 (nos. 3, 6, 7 and 13) completely recovered after the first treatment. Two cats (nos 12 and 16) showed a temporary worsening in the clinical status on day 14 (± 2) while their clinical score was similar to the pre-treatment evaluation after the second administration of Profender^®^. Specifically, cat no. 12 showed bronchovesicular sounds at day 0, bronchovesicular sounds, cough, tachypnoea, lethargy, and nasal and ocular discharge at day 14 (± 2), and cough and ocular and nasal discharge at day 28 (± 2). Cat no. 16 showed tachypnoea, dyspnea and bronchovesicular sounds at day 0 while at day 14 (± 2) pale mucous membranes were also evident and, at day 28 (± 2), the clinical presentation was similar to the pre-treatment evaluation. One cat in group T (no. 2) was apparently healthy at day 0, then its health started to worsen on day 14 (± 2) (i.e. tachypnoea was observed) but fully recovered on day 28 (± 2). One cat (no. 8) was apparently healthy throughout the study.

**Table 3:** Number of cats with clinical signs associated with troglostrongylosis

Clinical signs	Group	Day 0 *n* (%)	Day 14 *n* (%)	Day 28 *n* (%)	Day 42 *n* (%)	Day 56 *n* (%)
Cough	T	3 (37.5)	1 (12.5)	1 (12.5)	0	0
	C	0	1 (12.5)	1 (12.5)	1 (12.5)	0
Pale mucous membranes	T	1 (12.5)	1 (12.5)	0	0	0
	C	0	0	1 (12.5)	2 (25.0)	0
Hyperthermia	T	0	0	0	0	0
	C	0	0	0	2 (25.0)	0
Ocular-nasal discharge	T	2 (25.0)	1 (12.5)	1 (12.5)	0	0
	C	1 (12.5)	2 (25.0)	2 (25.0)	3 (37.5)	1 (12.5)
Tachypnoea	T	2 (25.0)	3 (37.5)	1 (12.5)	0	0
	C	1 (12.5)	2 (25.0)	2 (25.0)	3 (37.5)	0
Dyspnoea	T	3 (37.5)	1 (12.5)	1 (12.5)	0	0
	C	0	1 (12.5)	1 (12.5)	3 (37.5)	0
Abnormal breath sounds on lung auscultation	T	4 (50.0)	2 (25.0)	1 (12.5)	0	0
	C	0	0	1 (12.5)	1 (12.5)	0
No. of cats with at least 1 sign	T	5 (62.5)	3 (37.5)	2 (25.0)	0	0
	C	2 (25.0)	3 (37.5)	3 (37.5)	3 (37.5)	1 (12.5)

*Note*: Cats of Group T (*n* = 8); cats of Group C (*n* = 8)

With regard to control cats, before treatment (day 28 ± 2), 3 (nos 4, 10 and 11) had clinical signs related to *T. brevior* (Table 3). In particular, one (no. 11) completely recovered after the first administration of Profender^®^ while another (no. 4) completely recovered after the second treatment. The clinical signs in the third (no. 10), characterized by ocular and nasal discharge before treatment, worsened after the first administration (i.e. pale mucous membranes, hyperthermia, tachypnoea and dyspnea were recorded at day 42 ± 2), while the clinical score was similar to the pre-treatment evaluation from the second administration until the study end.

Two cats in Group C (nos 1 and 9), that were apparently healthy before treatment, showed a worsened health status on day 42 (± 2) (i.e. tachypnoea, dyspnea and nasal and ocular discharge were observed in cat no. 1 and pallor of mucosae and hyperthermia were recorded in cat no. 9) and recovered completely after the second rescue treatment. Three cats in group C (nos 5, 14 and 15) were apparently healthy throughout the study.

## Discussion

Nematodes of the genus *Troglostrongylus* have, for a long time, been considered as only affiliated to wild felids [4, 5] but, in the last decade, reports have documented a possible spread of *T. brevior* in domestic cats of Mediterranean and eastern regions, i.e. Italy, Greece, Cyprus, Spain and Bulgaria [10, 17–20].

Feline troglostrongylosis poses important challenges in feline medicine practice. Clinical diagnosis is impossible due to the overlapping clinical signs with aelurostrongylosis and other common diseases of cats [12, 21]. Furthermore, the Baermann migration test may have some shortcomings (e.g. false negative results are possible during prepatency and/or due to intermittent larval shedding, repeated examinations are recommended, L1 identification requires a skillful operator) [2, 4]. Once a definitive diagnosis is obtained, efficacious and timely treatment is crucial to save the life of the infected cat, especially in the case of young animals [2, 3, 7, 9].

The present study showed that one or two administrations of Profender^®^ (2 weeks apart) are highly effective and safe in treating troglostrongylosis, as no adverse effects occurred after administration. Indeed, the worsening of the health status of some of the cats in this study is probably due to death of the nematodes with a subsequent inflammatory host response leading to acute signs. This suggests that a concomitant administration of antiinflammatory drugs could be of benefit, especially in severely infected cats. Further clinical studies are warranted to elucidate this aspect.

The results of this study fit with those of published clinical cases where Profender^®^ has been used for treating single cats with troglostrongylosis and also in mixed infections with other respiratory nematodes [12, 13]. Thus, Profender^®^ can be considered potentially effective for treating feline troglostrongylosis in feline clinical practice, although further studies are necessary to confirm its efficacy in larger cohorts of animals. The Profender^®^ spot-on label (as also Broadline^®^) claims treatment for *A. abstrusus*, thus it could be successfully used in treating mixed infections by both *A. abstrusus* and *T. brevior*, which are fairly common [7, 12, 13]. Profender^®^ treats various round- and tapeworms and the use is not restricted to cases where the cats are at risk of co-infections with ectoparasites, thus allowing a broader use when helminth treatment alone is indicated.

On the other hand, spot-on eprinomectin is 100% efficacious against L4 and adult *T. brevior* after a single application [10, 14, 22], while the activity of spot-on emodepside has been thus far shown only against adult stages. Although this product should be given twice, 2 weeks apart to achieve a 100% efficacy, a very high reduction of LPG is already achieved after one administration against *A. abstrusus* [16] and *T. brevior* ([12], present results).

The use of spot-on emodepside is safe in kittens aged ≥ 8 weeks, which are at frequent risk of *T. brevior* infection [2, 10, 13, 20]. No studies have, however, investigated the safety of Profender^®^ in younger kittens, but in a recent study two administrations of the product administered off label by a veterinary practitioner were efficacious and safe in treating troglostrongylosis parasitologically and clinically in a kitten aged ≤ 8 weeks [8]. In another study the product was applied to kittens at 4 weeks of age, without any adverse effects being reported [23]. If one considers the frequent vertical transmission of *T. brevior*, this product could be potentially used to prevent potential lactogenic infection in kitten litters [8], because the formulation is safe in pregnant and lactating queens and effective in the prevention of the vertical transmission of *Toxocara cati* [23, 24].

## Conclusions

For its safety and efficacy, Profender^®^ can be considered a suitable choice for the treatment of natural feline troglostrongylosis.

## Abbreviations

SPC: Summary of product characteristics
LPG: Larvae per gram of feces
L1: First-stage larvae
L3: Third-stage larvae

## References

[1] Gerichter CB (1949). Studies on the nematodes parasitic in the lungs of Felidae in Palestine. Parasitology.

[2] Di Cesare A, Veronesi F, Traversa D (2015). Felid lungworms and heartworms in Italy: more questions than answers?. Trends Parasitol.

[3] Brianti E, Gaglio G, Giannetto S, Annoscia G, Latrofa MS, Dantas-Torres F (2012). *Troglostrongylus brevior* and *Troglostrongylus subcrenatus* (Strongylida: Crenosomatidae) as agents of broncho-pulmonary infestation in domestic cats. Parasit Vectors.

[4] Traversa D, Di Cesare A (2013). Feline lungworms: what a dilemma. Trends Parasitol.

[5] Traversa D, Di Cesare A (2016). Diagnosis and management of lungworm infections in cats: cornerstones, dilemmas and new avenues. J Feline Med Surg.

[6] Brianti E, Gaglio G, Napoli E, Falsone L, Giannetto S, Latrofa MS (2013). Evidence for direct transmission of the cat lungworm *Troglostrongylus brevior* (Strongylida: Crenosomatidae). Parasitology.

[7] Di Cesare A, di Regalbono AF, Tessarin C, Seghetti M, Iorio R, Simonato G, Traversa D (2014). Mixed infection by *Aelurostrongylus abstrusus* and *Troglostrongylus brevior* in kittens from the same litter in Italy. Parasitol Res.

[8] Traversa D, Salda LD, Diakou A, Sforzato C, Romanucci M, di Regalbono AF (2018). Fatal patent troglostrongylosis in a litter of kittens. J Parasitol.

[9] Diakou A, Di Cesare A, Aeriniotaki T, Traversa D (2014). First report of *Troglostrongylus brevior* in a kitten in Greece. Parasitol Res.

[10] Giannelli A, Capelli G, Joachim A, Hinney B, Losson B, Kirkova Z (2017). Lungworms and gastrointestinal parasites of domestic cats: a European perspective. Int J Parasitol.

[11] Crisi PE, Traversa D, Di Cesare A, Luciani A, Civitella C, Santori D, Boari A (2015). Irreversible pulmonary hypertension associated with *Troglostrongylus brevior* infection in a kitten. Res Vet Sci.

[12] Crisi PE, Aste G, Traversa D, Di Cesare A, Febo E, Vignoli M (2017). Single and mixed feline lungworm infections: clinical, radiographic and therapeutic features of 26 cases (2013–2015). J Feline Med Surg.

[13] Di Cesare A, Iorio R, Crisi P, Paoletti B, Di Costanzo R, Dimitri CF, Traversa D (2015). Treatment of *Troglostrongylus brevior* (Metastrongyloidea, Crenosomatidae) in mixed lungworm infections using spot-on emodepside. J Feline Med Surg.

[14] Giannelli A, Brianti E, Varcasia A, Colella V, Tamponi C, Di Paola G (2015). Efficacy of Broadline^®^ spot-on against *Aelurostrongylus abstrusus* and *Troglostrongylus brevior* lungworms in naturally infected cats from Italy. Vet Parasitol.

[15] Traversa D, Milillo P, Di Cesare A, Lohr B, Iorio R, Pampurini F (2009). Efficacy and safety of emodepside 2.1%/praziquantel 8.6% spot-on formulation in the treatment of feline aelurostrongylosis. Parasitol Res.

[16] Böhm C, Wolken S, Schnyder M, Basso W, Deplazes P, Di Cesare A (2015). Efficacy of emodepside/praziquantel spot-on (Profender^®^) against adult *Aelurostrongylus abstrusus* nematodes in experimentally infected cats. Parasitol Res.

[17] Jefferies R, Vrhovec MG, Wallner N, Catalan DR (2010). *Aelurostrongylus abstrusus* and *Troglostrongylus* sp. (Nematoda: Metastrongyloidea) infections in cats inhabiting Ibiza, Spain. Vet Parasitol.

[18] Di Cesare A, Veronesi F, Grillotti E, Manzocchi S, Perrucci S, Beraldo P (2015). Respiratory nematodes in cat populations of Italy. Parasitol Res.

[19] Diakou A, Di Cesare A, Barros LA, Morelli S, Halos L, Beugnet F, Traversa D (2015). Occurrence of *Aelurostrongylus abstrusus* and *Troglostrongylus brevior* in domestic cats in Greece. Parasit Vectors.

[20] Diakou A, Sofroniou D, Di Cesare A, Kokkinos P, Traversa D (2017). Occurrence and zoonotic potential of endoparasites in cats of Cyprus and a new distribution area for *Troglostrongylus brevior*. Parasitol Res.

[21] Foster SF, Martin P (2011). Lower respiratory tract infections in cats: reaching beyond empirical therapy. J Feline Med Surg.

[22] European Medicine Agency (2017). CVMP assessment report for Broadline Type II variation (EMEA/V/C/002700/II/0013).

[23] Wolken S, Schaper R, Mencke N, Kraemer F, Schnieder T (2009). Treatment and prevention of vertical transmission of *Toxocara cati* in cats with an emodepside/praziquantel spot-on formulation. Parasitol Res.

[24] Böhm C, Petry G, Schaper R, Wolken S, Strube C (2015). Prevention of lactogenic *Toxocara cati* infections in kittens by application of an emodepside/praziquantel spot-on (Profender^®^) to the pregnant queen. Parasitol Res.

